# Novel landscape of HLA‐G isoforms expressed in clear cell renal cell carcinoma patients

**DOI:** 10.1002/1878-0261.12119

**Published:** 2017-09-13

**Authors:** Diana Tronik‐Le Roux, Julie Renard, Jérôme Vérine, Victor Renault, Emmanuel Tubacher, Joel LeMaoult, Nathalie Rouas‐Freiss, Jean‐François Deleuze, François Desgrandschamps, Edgardo D. Carosella

**Affiliations:** ^1^ Commissariat à l'Energie Atomique et aux Energies Alternatives (CEA) Direction de la Recherche Fondamentale (DRF) Service de Recherche en Hemato‐Immunologie (SRHI) Paris France; ^2^ UMR_E5 IUH Hôpital Saint‐Louis Universite Paris Diderot Sorbonne Paris Cite France; ^3^ Service d'Anatomo‐Pathologie AP‐HP, Hôpital Saint‐Louis Paris France; ^4^ Centre d'Etudes du Polymorphisme Humain Fondation Jean Dausset Paris France; ^5^ Centre National de Génotypage Institut de Génomique CEA Evry France; ^6^ Service d'Urologie AP‐HP, Hôpital Saint‐Louis Paris France

**Keywords:** clear cell renal cell carcinoma, human leukocyte antigen G, immune checkpoints, isoforms, RNA sequencing, transcriptome

## Abstract

Immune checkpoints are powerful inhibitory molecules that promote tumor survival. Their blockade is now recognized as providing effective therapeutic benefit against cancer. Human leukocyte antigen G (HLA‐G), a recently identified immune checkpoint, has been detected in many types of primary tumors and metastases, in malignant effusions as well as on tumor‐infiltrating cells, particularly in patients with clear cell renal cell carcinoma (ccRCC). Here, in order to define a possible anticancer therapy, we used a molecular approach based on an unbiased strategy that combines transcriptome determination and immunohistochemical labeling, to analyze in‐depth the HLA‐G isoforms expressed in these tumors. We found that the expression of HLA‐G is highly variable among tumors and distinct areas of the same tumor, testifying a marked inter‐ and intratumor heterogeneity. Moreover, our results generate an inventory of novel HLA‐G isoforms which includes spliced forms that have an extended 5′‐region and lack the transmembrane and alpha‐1 domains. So far, these isoforms could not be detected by any method available and their assessment may improve the procedure by which tumors are analyzed. Collectively, our approach provides the first extensive portrait of HLA‐G in ccRCC and reveals data that should prove suitable for the tailoring of future clinical applications.

**Abbreviations**
CAGEKID: CAncer GEnome of the KIDney
ccRCC: clear cell renal cell carcinoma
H&E: hematoxylin and eosin
HLA‐G: human leukocyte antigen G
ILT: immunoglobulin‐like transcript
IMGT: international ImMunoGeneTics Project
KIR2DL4: killer cell immunoglobulin‐like receptor, two Ig domains, long cytoplasmic tail 4
LILRB: leukocyte immunoglobulin‐like receptor subfamily B
NCBI: National Center for Biotechnology Information
NGS: next‐generation sequencing
PDE5A: phosphodiesterase 5A
RNAseq: RNA sequencing
VEGF: vascular endothelial growth factor

## 1 Introduction

Immune checkpoints are crucial for the maintenance of self‐tolerance and for the modulation of immune responses in order to minimize tissue damage. Checkpoints result from the interaction between inhibitory ligand molecules and their receptors present on immune cells, mainly T cells. Tumor cells upregulate checkpoint ligands and take advantage of these associations to evade antitumor immunity, grow, and disseminate (Weber, 2010). At present, the administration of immune checkpoint inhibitors proved to be clinically successful in reestablishing immune function, thereby transforming radically cancer treatment (Postow *et al*., 2015). The interest of studying these molecules is even greater considering recent reports supporting the notion that immune checkpoint blockade may cooperate with commonly used chemotherapy cytotoxic drugs or radiation, to increase the effectiveness of therapeutic protocols (Tinhofer *et al*., 2016a,b).

A new immune checkpoint has recently been described: the nonclassical class I molecule human leukocyte antigen G (HLA‐G; Carosella *et al*., 2015). This molecule was first described to play a crucial role in the maintenance of pregnancy (Rouas‐Freiss *et al*., 1997) and was found constitutively expressed at the fetal–maternal interface in extravillous cytotrophoblasts. At present, HLA‐G has been found in most of the tumors analyzed. In particular, high incidence of HLA‐G expression has been reported in clear cell renal cell carcinoma (ccRCC; Bukur *et al*., 2003; Ibrahim *et al*., 2001), which is the most common human renal malignancy (Brugarolas, 2014). The role of HLA‐G as an immune checkpoint allowing tumor escape has been demonstrated in murine models (Agaugue *et al*., 2011; Loumagne *et al*., 2014).

Human leukocyte antigen G has a broader inhibitory effect than any other checkpoint as it can inhibit all steps of antitumor responses by acting on natural killer (NK) cells, B lymphocytes, T lymphocytes, and antigen‐presenting cells through direct interaction with the inhibitory receptor ILT2/CD85j/LILRB1 (ILT2), expressed by all monocytes, B cells, some NK cells, and T cells; ILT4/CD85d/LILRB2 (ILT4) receptor, expressed by monocytes and dendritic cells; and the KIR2DL4/CD158d (KIR2DL4) receptor that is expressed only in a subset cells of NK decidual cells (Carosella *et al*., 2015).

**Figure 1:**
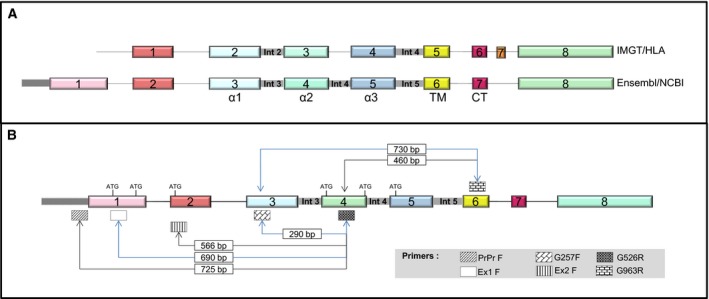
Schematic representation of the structure of the HLA‐G gene. (A) IMGT/HLA nomenclature (top) and Ensembl database (bottom). Numbers represent exons and the domains of the HLA‐G protein are shown underneath. TM, transmembrane; CT, cytoplasmic tail. (B) Localization of primers used for the different RT‐PCR strategies. Sizes, in bp, for specific amplicons and the translation initiation codons are indicated.

Even though HLA‐G has the makings of a major therapeutic target, several points have to be considered before its clinical use. One of these concerns the precise identification of isoforms that can be produced from the *HLA‐G* gene. Until now, most literature reports cover work on the membrane‐bound protein HLA‐G1. Still, smaller isoforms can be generated by alternative splicing (Ishitani and Geraghty, 1992). These may lack the alpha 2 domain, the alpha 3 domain, or both simultaneously (HLA‐G2, HLA‐G4, or HLA‐G3, respectively). The absence of the alpha 3 domain should prevent the binding to the receptors ILT4 or ILT2. Soluble proteins (HLA‐G5, HLA‐G6, and HLA‐G7) are also produced from the same gene following premature translation arrest at stop codons introduced by the retention of two introns (Fujii *et al*., 1994). This prevents the synthesis of the hydrophobic transmembrane domain which anchors the protein to the membrane. All seven reported HLA‐G isoforms have a similar translation start site and no distinct functional roles have yet been proposed (Riteau *et al*., 2001). In addition, there is still a discrepancy in numbering the exons of the HLA‐G gene. According to the Ensembl database, the HLA‐G gene might possess a supplementary exon at the 5′‐end that is absent from the IMGT/HLA database (Fig. 1A). The transcription of this supplementary exon would modify the size of the 5′‐UTRs and the location of the promoter. This may alter the regulation of the gene, by modifying the binding of regulatory proteins and/or miRNA. Moreover, the alternative exon selection might produce new protein variants that might potentially redefine cell fate and cancer progression.

For *HLA‐G*, as for most genes in the human genome, the complete annotation of all associated transcripts is still far from being exhaustive and identifying the complete set of isoforms that are generated remains a great challenge. RNA sequencing (RNAseq) analysis revealed, by mapping sequence reads to exon–exon junctions, that 92–94% of human genes undergo alternative splicing (Wang *et al*., 2008). Most of these isoforms vary in length, include introns, and even have very different amino acid sequences because splicing events can alter the translational reading frame of the differentially spliced mRNA. Moreover, they can have related, distinct, or even opposing functions.

Here, in order to define a possible HLA‐G based anticancer therapy, we performed an in‐depth analysis of ccRCC samples on the basis of immunohistochemical analysis and deep sequencing. Our results reveal a highly heterogeneous expression of HLA‐G among tumors, among distinct tumor areas of the same tumor, and in subcellular location of HLA‐G‐expressing tumor cells. In addition, we also provide an inventory of HLA‐G isoforms which includes the identification of still unreported forms. Altogether, our findings provide the first extensive portrait of HLA‐G in ccRCC which may impact the tailoring of future clinical applications.

## 2 Materials and methods

### 2.1 Tumor and patients

All patients of this study underwent a radical nephrectomy for ccRCC as first therapeutic intervention in the urology department of Saint‐Louis Hospital (Paris, France) from November 2014 to April 2015. The median tumor size was of 50 mm (range, 35–175). According to the 2010 primary tumor TNM classification, these tumors were classified as pT1a (patient 6), pT1b (patients 1, 3, and 8), and pT3a (patients 2, 4, 5, and 7). Two patients (patients 2 and 4) had visceral metastases at presentation. All these renal tumors were classified as ccRCC by an experienced uropathologist according to the World Health Organization classification of tumors of the kidney (Moch *et al*., 2016). All patients that participated in this study gave their free and informed writing consent. The study was approved by the institutional review boards of Saint‐Louis Hospital, Paris.

### 2.2 Tumor specimen processing

For each tumor and according to the tumor size, we isolated between 3 and 10 samples of 10 × 5 × 5 mm, representing the spatial extent and macroscopic intratumor heterogeneity. Half of each sample was snap‐frozen in liquid nitrogen within 1 h of clamping of the renal artery and the other half was used to perform histological analysis and was documented by photography. Regions that did not contain tumor cells on histopathological examination were also isolated as controls.

### 2.3 Immunohistochemistry

An immunohistochemical study was performed for each tumor on 4‐μm‐thick, formalin‐fixed, and paraffin‐embedded tumor tissue sections. The following murine antibodies were used: 4H84, an IgG1 recognizing an epitope located into the alpha 1 domain common to all HLA‐G isoforms (dilution 1/200; Santa Cruz Biotechnology, Santa Cruz, CA, USA), and two antibodies 5A6G7 and 2A12 recognizing the epitope encoded by the retained intron 5 (Ensembl database) present in soluble HLA‐G5 and ‐G6 isoforms (dilution 1/100, Exbio antibodies; Exbio, Praha, Czech Republic). The staining was performed on automated slide stainers from Roche (BenchMark ULTRA system, Tucson, AZ, USA) using the OptiView DAB IHC Detection Kit (Roche, Basel, Switzerland), Cell Conditioning 1 short or standard antigen retrieval, an antibody incubation time of 32 min at 37 °C, ultraWash procedure, counterstaining with hematoxylin II for 4 min, and bluing reagent for 8 min. Positive and negative controls gave appropriate results for each procedure.

The immunohistochemical analyses were performed by the uropathologist using a BX51 microscope (Olympus France S.A.S, Rungis, France). Each immunostaining was scored on the basis of membranous and/or cytoplasmic staining by both intensity of staining as negative, weak, moderate, or strong and distribution of staining as negative (0% of tumor area), minimal (0–10% of tumor area), focal (< 50% of tumor area), or diffuse (> 50% of tumor area). A trophoblastic tissue was used as the positive control, and isotype‐specific immunoglobulins were used for negative controls with each run.

### 2.4 Trophoblast sample preparation

Trophoblastic tissues were obtained from abortions (< 3 months of pregnancy). After mechanical dissociation, the samples were preserved in Trizol™ Reagent (LifeTechnologies, Carlsbad, CA, USA; ref. 15596‐026) at −80 °C until RNA extraction using the protocol described below.

### 2.5 RNA extraction

Total RNA was isolated from tissue sections manually crushed in Trizol™ Reagent (LifeTechnologies; ref. 15596026). After chloroform separation, the RNA was purified using miRNeasy mini Kit (Qiagen, Hilden, Germany; ref. 217004) according to the manufacturer's instruction, with a DNase treatment extra step (Qiagen; ref. 79254). The RNA purity and concentration was assessed using a Nanodrop spectrophotometer (Nanodrop, ThermoFisher Scientific, Waltham, MA, USA) and the Agilent 2100 Bioanalyzer System (Agilent, Santa Clara, CA, USA). RNA integrity number values were mostly > 8.

### 2.6 RT‐PCR

Reverse transcription of RNA into cDNA was performed using GoScript Reverse Transcriptase kit (Promega, Madison, WI, USA ref. A5001) with a MasterCycler Pro S thermocycler (Eppendorf, Hamburg, Germany). The PCR were carried out in a final volume of 10 μL, containing 2 μL of cDNA template, using an AmpliTaq polymerase from LifeTech (Ref. N80800166). For amplification, 40 cycles (at 94 °C for 30 s, 55 or 60 °C for 30 s, and 72 °C for 30 s) were conducted. HLA‐G and actin (ATCB) primers are described in Table 1. ATCB amplification was performed as control in all the experiments. The PCR amplification product was mixed with 6× loading dye (Promega; ref. G1881) and analyzed on 2% agarose gel stained with 2 μL of ethidium bromide at 1 mg·mL^−1^ for 100 mL of agarose gel. The molecular weight marker used was 1 kb plus DNA ladder from Invitrogen, Carlsbad, CA, USA (Ref. 10787018). Imaging was performed using a ChemiDoc XRS System (Bio‐Rad, Hercules, CA, USA), and interpretation using imagelab software (Bio‐Rad).

**Table 1:** PCR primers for RT‐PCR experiments

Genes	Sequence (5′–3′)
PrPr F	5′‐GTAACATAGTGTGGTACTTTG‐3′
Ex1F	5′‐CCTGGACTCACACGGAAACT‐3′
Ex2F	5′‐GGACTCATTCTCCCCAGACG‐3′
257 F	5′‐GGAAGAGGAGACACGGAACA‐3′
257 R	5′‐TGTTCCGTGTCTCCTCTTCC‐3′
526 F	5′‐CCAATGTGGCTGAACAAAGG‐3′
526 R	5′‐CCTTTGTTCAGCCACATTGG‐3′
963 R	5′‐GCAGCTCCAGTGACTACAGC‐3′
Int1F	5′‐GGCCTCAAGCGTGGCTCTCA‐3′
Int3F	5′‐CCCAAGGCGCCTTTACCAAA‐3′
Int4R	5′‐CCACTGCCCCTGGTAC‐3′
Int5R	5′‐AGCCCTCACCACCGACC‐3′
ATCB F	5′‐TCCTGTGGCATCCACGAAACT‐3′
ATCB R	5′‐GAAGCATTTGCGGTGGACGAT‐3′

### 2.7 RNA sequencing

Indexed complementary DNA libraries were prepared from 1 μg of total RNA following the Illumina TRUSEQ protocol. Average size of the AMPure XP beads (Beckman Coulter, Brea, CA, USA) purified PCR products was 275 bp. The paired‐end 150‐bp reads sequencing of the transcriptome was performed on equimolar pools of four cDNA libraries on a NextSeq 500 (Illumina, San Diego, CA, USA).

### 2.8 High‐throughput analysis of HLA‐G isoforms

The Ensembl nomenclature will be used throughout the text. Short reads from next‐generation sequencing (NGS) were mapped to human Reference Genome NCBI Hg19 using tophat2 aligner (Kim *et al*., 2013). Low‐quality mapping reads were filtered out from alignment files and the reads mapping to the HLA‐G locus were extracted using samtools (Li *et al*., 2009). Intron‐retained detection was performed by selecting reads overlapping an intron and one of the surrounding exons, and retention for an intron was assessed only when we detected reads overlapping both 5′‐ and 3′‐flanking exons. Exon skipping detection was performed by analyzing reads presenting split mapping, searching for discontinuity in the order of mapped exons: A read that is mapped to exon the end of 4 and start of exon 6 but is not mapped to exon 5, presents a skipping of exon 5. Each read subset was visually validated with integrative genomics viewer (Robinson *et al*., 2011). For the retention of intron *n*, the percentage of reads $p_{i}^{n}$ supporting the event is calculated as the ratio between the reads supporting the events (reads at junction exon *n*/intron *n*, internal intronic reads on intron *n,* and reads at junction intron *n*/exon *n* + 1) and the total number of reads spanning the region where the event occurs (the region starting from the junction between exon *n* and intron *n* to the junction between intron *n* and exon *n* + 1): Let *R*(*i*) be the number of reads strictly in region *i* (the reads are only in region *i* and do not overlap with other regions) and *R*(*i, j*) be the number of reads overlapping both regions *i* and *j*. Let *S*(*i*) be the number of reads supporting a skipping of exon *i* (reads overlapping exon *n* and exon *m* where *m* > *n* + 1). The number of reads supporting the retention of intron *n* is thus IR_*n*_ = *R*(exon_*n*_, intron_*n*_) + *R*(intron_*n*_) + *R*(intron_*n*_, exon_*n* + 1_). The total number of reads in the region of the retention of intron *n* is *T*_*n*_ = IR_*n*_ + *R*(exon_*n*_, exon_*n* + 1_) + *S*(*n*); $p_{i}^{n}$ is thus given by $p_{i}^{n}={\text{IR}}_{n}/T_{n}$.

For the skipping of exon *n*, the percentage of reads $p_{e}^{n}$ supporting the event is given by $p_{e}^{n}=S\left(n\right)/T_{n}$. Analysis of potential biases was assessed by reprocessing the data using the bwa aligner (bwa mem option; Li and Durbin, 2009).

## 3 Results

### 3.1 Marked subcellular heterogeneity of HLA‐G isoforms distribution in ccRCC

**Figure 2:**
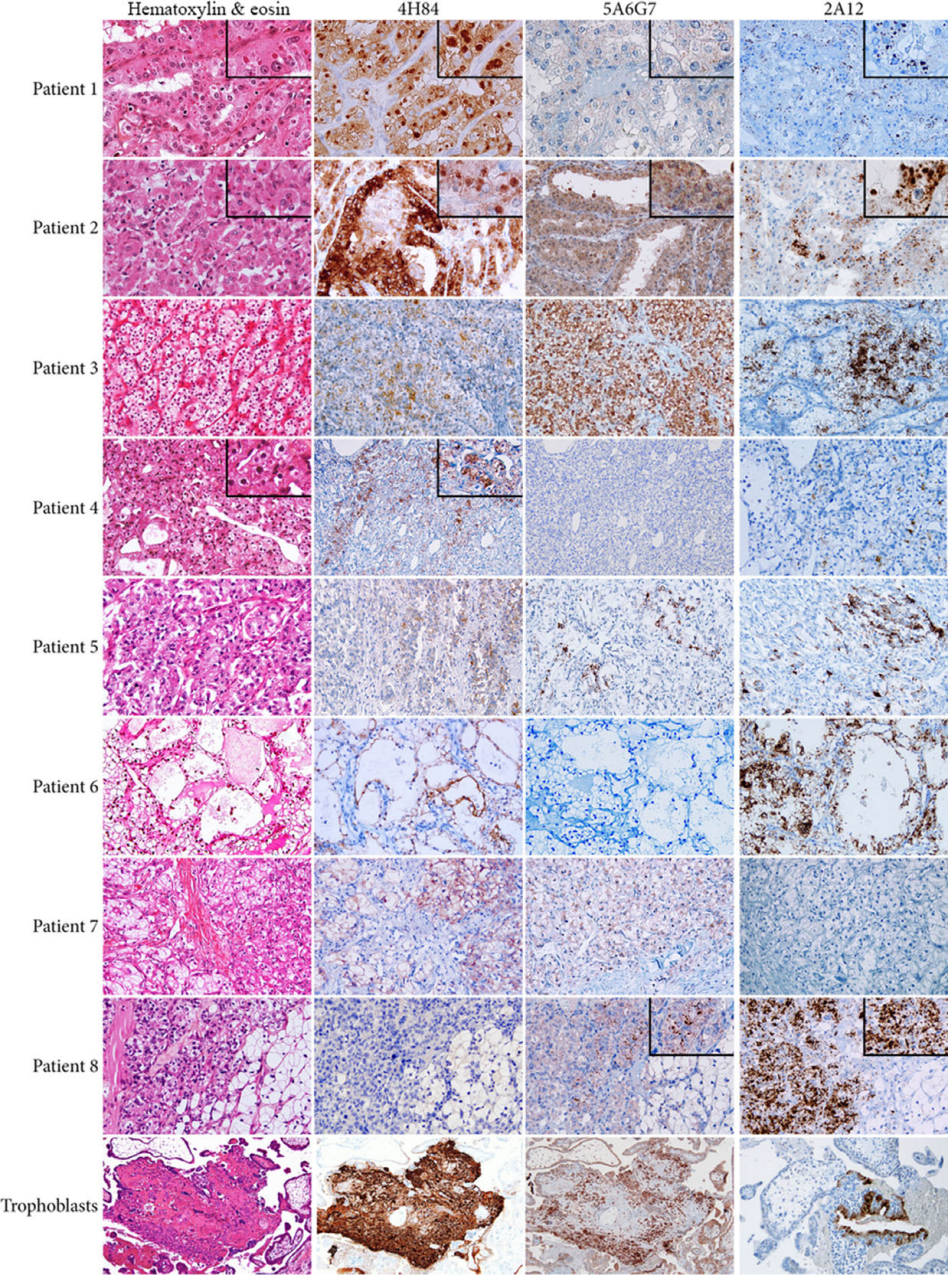
Differential morphologic and HLA‐G staining patterns of eight ccRCC included in this study. A trophoblastic tissue was used as positive control for immunohistochemical study (H&E and immunoperoxidase stains).

In order to consider HLA‐G as a potential target for cancer therapy, we ought to precisely determine how prevalent the expression of HLA‐G is in tumor cells derived from patients with ccRCC. To this end, we have isolated 3–10 sections for each tumor, according to the tumor size. Microscopy analysis performed on hematoxylin and eosin (H&E)‐stained slides confirmed a morphologic heterogeneity (Fig. 2, left panel), classically associated with ccRCC (Moch *et al*., 2016). We further dissected this heterogeneity by immunostaining with specific antibodies directed against HLA‐G: 4H84, which recognizes an epitope located into the alpha 1 domain common to all seven reported HLA‐G isoforms, and the antibody 5A6G7 that only recognizes soluble HLA‐G5 and HLA‐G6 isoforms. This antibody targets the amino acids encoded by the retained intron 5 (previously known as intron 4 according to the IMGT/HLA nomenclature). Trophoblastic cells, which express HLA‐G at high levels, were used as positive controls.

Even though all tumors expressed HLA‐G in at least one area, this expression was distinct between and inside tumors. Tumors of patients 1 and 2 showed a strong immunostaining with 4H84 antibody in all regions. The staining was membranous and cytoplasmic (Fig. 2). Noteworthily, an additional very strong staining of hyaline globules located in the cytoplasm of the tumor cells was also detected. These hyaline globules were well visible on H&E slides and constituted a very uncommon aspect of tumor cells (Krishnan and Truong, 2002). On the other hand, using the 5A6G7 antibody, a weak or moderate granular cytoplasmic immunostaining was noticed in the cytoplasm, but not in hyaline globules. The expression of HLA‐G in tumors from other patients was very different: Tumors of patients 6 and 7 presented a diffuse but moderate membrane immunostaining with 4H84 antibody. These two tumors showed no (patient 6) or weak and focal (patient 7) granular intracytoplasmic immunostaining with 5A6G7, which denoted the absence of soluble proteins HLA‐G5 and HLA‐G6. In two other tumors (patients 4 and 5), the expression of HLA‐G evaluated by 4H84 antibody was noted in small microscopic areas of only one tumor region. Of note, the only HLA‐G‐positive area of patient 4's tumor corresponds precisely to intracytoplasmic hyaline globules. No stain was observed in any other region of the tumor.

The immunostaining profiles of tumor cells of patients 3 and 8 were unexpected. No immunostaining was detected with the 4H84 antibody that labels all the reported HLA‐G isoforms. The lack of labeling of tumor sections with this antibody normally accounts for the absence of HLA‐G expression. However, a diffuse and strong granular intracytoplasmic 5A6G7 immunostaining and a diffuse, thin, and granular intracytoplasmic immunostaining were observed in tumor cells of patients 3 and 8, respectively. This was unpredictable considering our current knowledge on the structure of the seven reported HLA‐G isoforms as they all contain the alpha 1 domain recognized by the 4H84 antibody. To try to better understand these differences, we have performed a similar analysis using an antibody that also recognizes the epitope encoded by the retained intron 5 (Ensembl database) present in soluble HLA‐G5 and ‐G6 isoforms named 2A12 (Fig. 2, right column). The results revealed different and unanticipated immunostaining patterns, notably the labeling of hyaline globules in patients 1 and 2.

Together, the results of the immunohistochemical study clearly demonstrate intra‐ and interheterogeneity of HLA‐G expression in ccRCC tumors. However, some immunostaining patterns were unexpected within the boundaries of our prevailing knowledge on the structure of HLA‐G isoforms.

### 3.2 Survey of HLA‐G1 transcripts expressed in ccRCC

**Figure 3:**
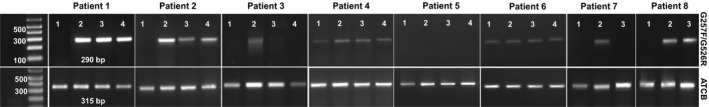
Expression of HLA‐G1 in ccRCC patients. RNA were subjected to RT‐PCR using the HLA‐G1‐specific primers G257F and G526R (upper panels) and ACTB primers as controls (lower panels). Lanes 1: adjacent nontumor region except for tumors of patients 6 and 8. Lanes 2, 3, and 4: different tumor areas. For patients 6 and 8, all regions shown correspond to tumor areas as partial nephrectomies were performed and adjacent tumor regions were not available. M: 100‐bp size marker.

To gain a better insight into the HLA‐G isoforms that are expressed in ccRCC and clarify the results of the immunohistochemical analysis, a survey of HLA‐G isoform diversity was further assessed by RT‐PCR. The tumor sections of the eight patients studied above were amplified with the well‐known G257F and G526R primers (Paul *et al*., 2000b) schematically represented in Fig. 1B (to avoid misunderstandings, the Ensembl nomenclature will be used throughout the text). These primers amplify a region that contains the epitope recognized by the 4H84 antibody. Amplification of actin mRNA was performed for each sample as control. A predicted band of 290 bp, specific for the amplification of HLA‐G1 transcripts, was found in all tumor sections for patients 1, 2, and 6, whereas this band was only detected in one or two regions of tumors of other patients (Fig. 3). No amplification products were detected in nontumoral adjacent tissues under these conditions. As the sequences of the different isoforms are highly similar and these RT‐PCR conditions do not allow the identification of other isoforms like HLA‐G2, ‐G3, ‐G6, or ‐G7 which lack exon 4, the target of primer G526, we undertook a large‐scale study by RNAseq in order to provide a comprehensive picture of isoforms expressed in ccRCC.

### 3.3 RNAseq reveals unannotated HLA‐G transcripts

RNAseq technology provides the most powerful method to analyze expressed isoforms, offering the opportunity to detect alternative splicing events and unannotated transcripts which are essential for understanding development and disease mechanisms in a species (Wang *et al*., 2009).

As a first look, we have undertaken the sequencing of four representative samples at a very high depth of coverage (depth > 300×). Reads were aligned and quantified according to the Ensembl 70 (GRCh37.p8) reference annotation as described in Materials and methods. Alternative spliced isoforms were mainly categorized into two major groups: exon skipping and intron retention, in which a single exon or intron is alternatively spliced or included out of the mature message, respectively.

To verify whether the HLA‐G expression patterns of patients with ccRCC described above constitute a representative subset of general profiles found in patients with ccRCC, we have compared our results to those obtained for the ‘CAncer GEnome of the KIDney’ (CAGEKID) cohort, which includes a hundred patients with ccRCC that were treated in four different European countries (Czech Republic, United Kingdom, Romania, and Russia). The data that have been generated constitute a high‐quality resource that allowed detecting alternative splicing events with high accuracy (Scelo *et al*., 2014). To assess whether common factors such as the choice of the aligner for RNAseq data might potentially bias our analysis, we use two different aligners, tophat2 and bwa mem. The results confirmed that the data aligned with tophat2 or bwa mem produce similar results (Data S1, Table S1 and Fig. S1). In addition, we assess whether other HLA loci might bias the results. To this end, we aligned the whole HLA‐G sequence extracted from human genome hg19 against hg19 using blastn 2.2.26 (blast+ 2.6.0, National Center for Biotechnology Information, U.S. National Library of Medicine, Rockville Pike, Bethesda, MD, USA) with default options. We found that using only high‐quality mapped reads (MAPQ ≥ 30), the best match is always HLA‐G. Secondary hits have at most 90 bp (in the 150‐bp reads) that match exactly (no gap, no mismatch) with other HLA sequences. For the whole locus of HLA‐G and exons 1, 6, 7, 8 as well as introns 3 and 5, no significant match was found with other HLA loci. For the other exons/introns, the longest alignment (HSP) does not exceed 70 bp (Data S2, Tables S2 and S3). Further, the count of reads at the individual level showed a great similarity between the expression profiles of HLA‐G transcripts found in our small cohort of patients with ccRCC and that of CAGEKID. These results are summarized on Tables 2 and 3 and will be discussed more thoroughly in the following sections.

**Table 2:** Number of reads for all observed HLA‐G splicing events in ccRCC samples

	Patient 1	Patient 3	Patient 4	Patient 5	B00E4I3	B00E4IS
#Reads at HLA‐G locus	4925	2709	1037	129	6503	5462
#Reads q30 at HLA‐G locus (mean)	4495	1552	110	67	5692	4605
Exon1 total reads	11	4	0	0	0	0
Exon2 total reads	243	30	2	0	238	221
Exon3 total reads	1594	410	13	5	1126	1376
Exon4 total reads	2364	525	50	17	2141	2104
Exon5 total reads	1957	687	67	52	4279	2892
Exon6 total reads	1113	315	6	6	2137	1493
Exon7 total reads	896	454	0	9	1002	743
Exon8 total reads	2214	1190	23	41	3245	2438
Retention of intron 1	22	4	0	0	20	6
Retention of intron 2	2	0	0	0	4	1
Retention of intron 3	102	25	2	2	19	49
Retention of intron 4	82	15	2	0	11	23
Retention of intron 5	154	27	8	0	184	95
Retention of intron 6	12	4	0	0	6	5
Retention of intron 7	74	31	0	0	21	37
Skipping of exon 4	5	1	0	0	29	13
Skipping of exon 5	0	0	0	0	3	0
Skipping of exon 6	4	2	0	0	11	9
Skipping of exon 7	32	6	0	0	81	41
Skipping of exon 4 and 5	2	3	0	0	6	6
Skipping of exon 4,5,6 and 7	0	0	0	0	0	0
Skipping of exon 4,5 and 7	0	0	0	0	0	0
Skipping of exon 5,6 and 7	0	0	0	0	0	0
Skipping of exon 6 and 7	5	2	0	0	24	5
Raw count of reads start exon2	132	15	0	0	64	106
Raw count of reads start exon3	4	0	0	0	0	4
Raw count of reads start exon4	72	30	6	0	101	96
Raw count of reads start exon5	8	2	0	0	15	6

Patients 1, 3, 4 and 5 are representative samples selected for exploring the diversity of HLA‐G isoforms. B00E4I3 and B00E4IS are the two samples with the highest HLA‐G expression within the CAGEKID (CAncer GEnome of the KIDney) (Scelo *et al*., 2014).

**Table 3:** Percentage of transcripts for each splicing event observed

Alternative splicing events	% Overall samples	Median	% Overall samples CAGEKID	Median CAGEKID
Retention of intron 1	50	100	85.71	100
Retention of intron 2	25	0	6.49	0
Retention of intron 3	75	55.02	89.61	18.03
Retention of intron 4	100	30.77	88.31	11.94
Retention of intron 5	75	31.30	93.51	13.98
Retention of intron 6	100	75.68	100	94.44
Retention of intron 7	100	57.70	88.31	45.45
Skipping of exon 4	0	0	50.65	0.47
Skipping of exon 6	0	0.32	71.43	5.13
Skipping of exon 7	50	1.35	84.42	43.75
Skipping of exon 6 and 7	0	0	68.83	8.70

Percentage of overall samples is the percentage of samples presenting the event. The last two columns are the same metrics calculated for 77 CAGEKID samples expressing HLA‐G.

### 3.4 Undescribed intron retention events in expressed HLA‐G transcripts

Intron retention is the rarest type of alternative splicing in mammals and accounts for only approximately 3% of alternate transcripts (Wong *et al*., 2016). So far, only the retention of intron 3 or intron 5 (previously known as intron 2 and intron 4, according to IMGT/HLA nomenclature) was reported in the literature for HLA‐G transcripts. Transcripts that retain intron 3 encode HLA‐G7 (Paul *et al*., 2000a), and those retaining intron 5 encode HLA‐G5 and HLA‐G6 (Fujii *et al*., 1994).

**Figure 4:**
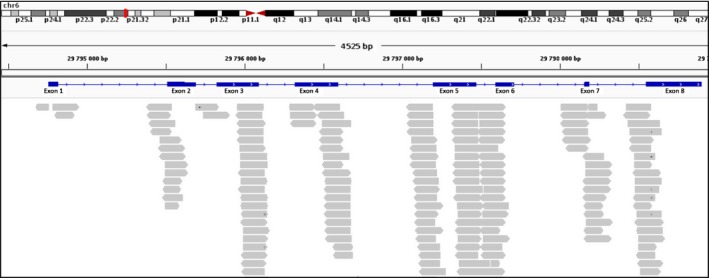
Details of intron retention events found in HLA‐G transcripts. Only reads spanning intron–exon junctions have been considered. Reads corresponding exclusively to intron sequences were discarded.

In our RNAseq analysis, introns subsumed by an exon were labeled as retained. The results, represented graphically on Fig. 4 and summarized in Table 2, showed that reads representing the retention of introns 3 and 5 were the most abundant. In addition, the data support a number of overall new findings that originate from the retention of four additional introns: 1, 4, 6, and 7. To confirm that reads supporting intron‐retained events are truly HLA‐G intron‐supporting reads, we extracted at the HLA‐G locus full intronic reads along with reads overlapping an exon and intron at the HLA‐G locus. We then aligned these reads (150 bp) against the IMGT/HLA reference which contains all HLA alleles, using blast 2.2.29+ with highly stringent parameters (100% of homology, no gap and minimum Expect value of 1e‐40). No alignment was found to match other HLA loci better than HLA‐G (Data S2 and Table S4).

**Figure 5:**
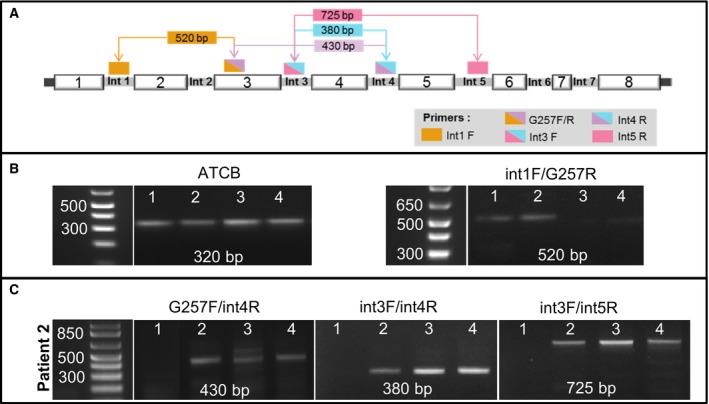
Molecular validation of main intron retention events. (A) Diagrammatic representation of the RT‐PCR strategy developed to amplify retained introns. (B) Results of the RT‐PCR analysis using actin primers as control for the absence of genomic DNA (left) and Int1 and G257R primers to detect the presence of intron 1 (right). The band of 523 bp reveals the absence of intron 2, which would produce a band of 649 bp (C) HLA‐G transcripts that retain only intron 4 (left panel) or HLA‐G transcripts that retain several introns simultaneously (middle and right panels).

To experimentally validate the expression of intron‐retained transcripts, we first looked for the presence of transcripts containing the intron 1. To this end, we performed RT‐PCR amplifications using a strategy described in Fig. 5. First, primer that targets intron 1 (Int1F) was used in combination with G257R, the reverse primer of G257F (Paul *et al*., 2000b). As the presence of introns may be due to contaminating endogenous genomic DNA, all samples were amplified in parallel with actin‐specific primers located in two different exons. The expected size for the amplification of cDNA derived from mRNA is 320 bp, whereas that of genomic DNA is 560 bp. The results show only the amplification of a 320‐bp fragment in all samples, demonstrating the absence of genomic contamination (Fig. 5B, left panel). In view of this result, we further amplified tumor samples using primers Int1F and G257R. An amplified band of the expected size (521 bp) was obtained, consistent with the presence of intron 1 in HLA‐G transcripts (Fig. 5B, right panel). This event was not reported before in the literature as the initiation of transcription of HLA‐G was solely assigned to exon 2 (Geraghty *et al*., 1987). We did not detect a PCR amplification band of 649 bp that would correspond to the concomitant retention of intron 2. This is consistent with the results of the RNAseq analysis showing that intron 2 is infrequently retained.

Further analysis was conducted to validate the retention of intron 4 (Fig. 5C). To this end, RT‐PCR was performed using primer G257F in combination with a primer that specifically targets intron 4 (named Int4R). Amplification with these primers generated a DNA fragment of 430 bp (Fig. 5C, left panel), demonstrating the presence of intron 4 in HLA‐G transcripts. The size of the amplified band is also consistent with the presence of a concomitant retention of intron 3. To further assess whether the same transcript might retain several introns simultaneously, we have performed a RT‐PCR amplification using primer Int3F (whose sequence is complementary to a region of intron 3) in combination with primer Int4R. The results reveal a DNA fragment of 380 bp, as expected for the retention of introns 3 and 4 in the same transcript (Fig. 5C, middle panel). In addition, amplification with Int3F and Int5R primers generated an amplified band of 725 bp (Fig. 5C, right panel). Of note, the size of this band corresponds to the retention of introns 3 and 5, excluding intron 4. These results clearly demonstrate that tumor samples might express transcripts that retain a single intron and others that retain several different introns which may vary from one transcript to the other. To our knowledge, these events were not previously described.

### 3.5 Novel HLA‐G transcripts with 5′‐extended end

**Figure 6:**
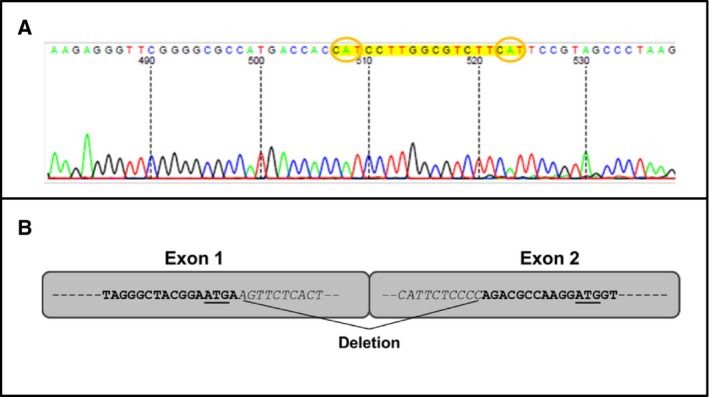
Identification of the 5′‐extended transcript HLA‐G1. (A) Details of the DNA sequence showing the reduced distance between the two ATGs. The sequencing was performed upward using the G526R primer. (B) Schematic representation of the 106‐bp deletion; the two ATGs are underlined.

The RNAseq data further revealed that some of the reads aligned on either side of exon 1 (Fig. 4). Transcripts that originate from this area were not previously reported. In fact, the structure of this region is still a matter of debate as information contained in the Ensembl database suggests that HLA‐G transcripts may be initiated at this exon, which is located 5′ of the exon 1 defined by IMGT/HLA nomenclature (Fig. 1A). The presence or absence of this exon may result in major modifications which include the promoter localization, the length of the 5′‐UTR, and the transcription/translation initiation site. We assess whether HLA‐G transcripts may be initiated in this exon or even upstream by RT‐PCR amplification. Two specific primers were designed: primer Ex1F, whose sequence is complementary to a region located in exon 1 (Ensembl database), and primer PrPr, whose sequence is complementary to a region located further upstream currently considered as the promoter region (schematically represented in Fig. 1B). RT‐PCR using these two upstream primers in combination with G526R produced two bands of expected sizes: 690 bp (for Ex1F‐G526R) and 725 bp (for PrPr‐G526R), respectively (data not shown). To verify the specificity of these fragments, amplified DNA samples were sequenced and nucleotide similarities were searched in public databases using blast. The results demonstrated a high degree of similarity with HLA‐G except for a deletion of 106‐bp fragment. Resulting from this deletion, the distance between the ATG located at the end of exon 1 and the one located in exon 2 was reduced from 118 to 12 bp (Fig. 6A). As a consequence, the 106‐bp deletion brings both ATG in frame. This may now allow the initiation of translation at the ATG located in the first exon and generate a protein that would have a 5′‐extended end of five additional amino acids (MKTPR). At present, the only translation initiation start site was attributed to the ATG located in exon 2 (which corresponds to exon 1 defined by IMGT/HLA nomenclature). This transcript was also found in some of the trophoblast samples tested but not all. This indicates that factors regulating its expression are still to be elucidated.

Altogether, these results are consistent with the existence of a novel HLA‐G transcript, named HLA‐G1L, having an extended 5′‐end, which might be coexpressed in trophoblasts and ccRCC tumor cells with previously reported HLA‐G isoforms.

### 3.6 Alternatively spliced exons potentially generate novel soluble HLA‐G isoforms

Exon skipping is one of the major forms of alternative splicing, which generates multiple mRNA isoforms differing in the precise combinations of their exon sequences. Here, we define an exon skipping event as a pairing between an exon‐containing form and an exon‐excluding form, occurring at the same exon and with the same flanking introns. The same exon may be involved in multiple exon skipping events.

**Figure 7:**
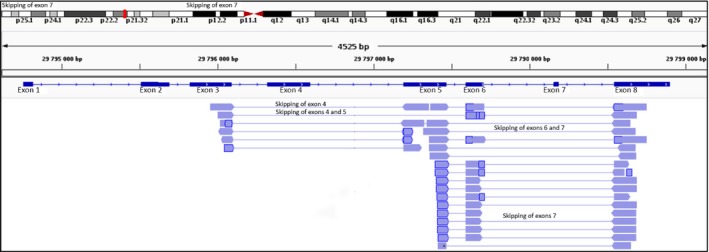
Mapping of RNAseq reads generated with tophat2 aligner showing main exon skipping events in HLA‐G (reads that did not show split mapping were hidden to improve readability).

For HLA‐G, only the skipping of exon 4 (HLA‐G2), exon 5 (HLA‐G4), or both simultaneously (HLA‐G3) was reported in the literature. In this study, aligned reads with tophat2 reveal the skipping of exons never uncovered before. The main skipping events are graphically represented in Fig. 7 and reported in Table 2. We also confirmed these results by using the bwa mem aligner (Fig. 1, Data S1, S2, Fig. S1 and Tables S1–S4). The highest read coverage was consistent with the skipping of exon 7 alone, which contains the stop codon of the protein. However, no major modifications are expected in the encoded protein lacking this exon as a supplementary in‐frame stop codon is found at the beginning of exon 8. Most importantly, skipping of exon 7 concomitantly to exon 6, which encodes the transmembrane domain, is highly relevant as their absence may generate isoforms that lack the transmembrane domain and the cytoplasmic tail and therefore would constitute still unreported soluble proteins.

**Figure 8:**
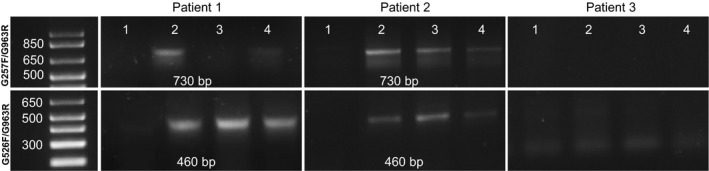
Expression of transcripts lacking exon 3 which encodes the alpha 1 domain and exon 6, the cytoplasmic domain. RT‐PCR amplification of RNA of tumors from patients 1, 2, and 3 with primers G257F/G963R and G526F/963R, respectively. Primer G257 is complementary to exon 3, G526 to exon 4, and G963 to exon 6. The cDNAs are the same as in Fig. 3, in which their integrity was shown by actin amplification.

When RT‐PCR was performed with primer G963R, whose sequence is complementary to a region of exon 6, no amplification products could be obtained in combination with the forward primer G257F (exon 3) or G256F (exon 4). However, an expected 290‐bp amplified fragment was generated when the primer G257F was used in combination with G526R. Together, these results are consistent with HLA‐G transcripts that possess exons 3 and 4 but lack exon 6 (Fig. 8). In addition, when these primers were used to analyze samples from patient 1, amplified bands were obtained using the primer combination G526F–G963R, whereas no amplification was detected using G257F–G963R, consistent with the expression of transcripts that lack exon 3.

### 3.7 Alternative spliced HLA‐G isoforms lack the alpha‐1 domain

Further analysis of RNAseq data reveals that some of the reads might be initiated at exon 4. This was determined by quantifying the raw count of reads within 20 bp upstream of the exon acceptor site. The predicted N‐terminal‐truncated protein would lack the peptide signal and the alpha 1 domain. To assess whether the translation into a protein might start in this region, we have examined the nucleotide sequence of exon 4. This analysis revealed the presence of an in‐frame ATG that might serve as a translation initiation codon. Our preliminary results (not shown) reveal that transcripts that lack the alpha‐1 domain may lack also the alpha‐2 domain and therefore encode only the alpha‐3 domain. Further work is needed to investigate whether this shortened transcript encodes functional proteins.

Notably, the expression of these isoforms may now provide a hypothesis on the differences in immunostaining patterns generated following the labeling of some tumor samples with 4H84 and antibodies that have been raised against soluble isoforms, which could not be explained previously within the boundaries of widespread knowledge on the structure of HLA‐G isoforms.

## 4 Discussion

During the last years, the advent of NGS technologies has greatly accelerated biological and medical research and dramatically changes our perception of cancer. In particular, their application to RNAseq analysis considerably expanded our knowledge on how the coding potential of eukaryotic genomes can be amplified by the use of alternative splicing of exons and introns of precursor messenger. Thus, this process provides an exceptional manner to bring forth, in a previously unimaginable manner, multiple protein isoforms having minor structural differences but altered biological properties, including their subcellular locations and functions (Wang *et al*., 2008). Accordingly, uncovering isoforms that are expressed in tumor cells might assuredly open new perspectives in the cancer research field, more specifically for use in early detection, diagnosis, and therapeutic strategy design.

In this study, we have shown that combining immunohistochemistry with transcriptome determination can be effective to provide a precise overview of structurally different HLA‐G isoforms expressed in tumors derived from patients with ccRCC. Our molecular survey has not only revealed a high intra‐ and intertumor heterogeneity regarding HLA‐G expression, but also highlighted distinct subcellular locations and, for the first time, bring out previously undescribed isoforms which might encode proteins internally deleted or having different N‐ or C‐terminal regions coexpressed in the same tumor.

Even though all the ccRCC tumors examined expressed HLA‐G in at least a small region of the tumor, as shown by the immunohistochemical analysis, largely dissimilar profiles were revealed. Tumor cells may or may not express one or several membrane‐bound and/or soluble forms, which can be scattered along the entire tumor or restricted to particular tumor areas. In some tumors, the presence of hyaline globules was noted which were found to express HLA‐G. These hyaline globules are barely described in ccRCC (Nayar *et al*., 2000) and their functional role has yet to be determined. Whether the high diversity of the HLA‐G expression level is a consequence of spatial separation and selection of subclones is still to be determined (Gerlinger *et al*., 2014).

Different immunostaining patterns were observed with antibodies 5A6G7 and 2A12. These two antibodies have been produced in the same manner from splenocytes of Balb/c mice immunized with an ovalbumin‐bound synthetic 21‐mer peptide (SKEGDGGIMSVRESRSLSEDL) derived from the carboxy‐terminal sequence of soluble HLA‐G5 and HLA‐G6 proteins. Therefore, it would have been expected that the 2A12 and 5A6G7 staining patterns would be the same. Although differences in immune labeling with these antibodies were previously seen (Larsen *et al*., 2011), this finding was not anticipated. This might be explained by (a) distinct epitopes recognized by these antibodies which, due to HLA‐G conformational changes or association with other proteins selectively present in some tumors, may no longer be accessible, and (b) novel HLA‐G spliced isoforms lacking either 5A6G7 or 2A12 epitope. Another puzzling result was the staining profile of patient 8's tumor. When using the 4H84 antibody (that recognizes all the reported HLA‐G isoforms), no stain was detectable, whereas a strong immunostaining was observed with 5A6G7 or 2A12 antibodies (that labels only HLA‐G5 and HLA‐G6). Altogether, these unexpected findings suggested the presence of still undescribed isoforms and prompted us to initiate a more extensive analysis at the molecular level.

Combining RNAseq and RT‐PCR analysis provided a highly improved view on the expression of HLA‐G isoforms. We describe, to our knowledge for the first time, HLA‐G transcripts that are initiated at exon 1. This exon 1 is not included in the IMGT/HLA‐G database. Although this sequence is listed on the Ensembl database (ENST00000376828.6), to our knowledge no articles have been published. Our results suggest that the ATG codon present at the end of exon 1 might be used as translation initiation site due to the absence of a 116‐bp region located 5′ of the ATG found in exon 2. This ATG was not previously considered suitable for the initiation of translation as it is out of frame with respect to the ATG present in exon 2, which has been considered as the sole possible translation initiation site. Resulting from this deletion, a reading frame shift occurs as the number of deleted nucleotides is not multiple of 3. This brings the two ATGs in frame, which allows to potentially encode a protein with an extended N‐terminal region of five amino acids.

If this protein would be actually synthesized *in vivo*, the consequences of the different hydrophobicity of the N‐terminal region would be multiple. On the one hand, it might modify not only the intracellular localization of the isoform, but also their conformation, kinetic properties, and the fate of other molecules. This would be the case of the dependence of HLA‐E expression on the HLA‐G‐derived signal peptide (Carosella *et al*., 2000). Even though other peptides that differ in length and sequence might also allow the expression of HLA‐E at the surface of the cell, these peptides may prevent HLA‐E interaction with its inhibitory receptor NKG2A, resulting in high increase in target cell lysis (Hoare *et al*., 2008). Indeed, HLA‐E is the sole ligand for the NKG2AB/CD94 receptor on NK cells; conformational changes in the peptide that is bound to HLA‐E will have an impact on the recognition by the NKG2A/CD94 receptor (Kraemer *et al*., 2015).

In addition, another very important consequence of the presence of this 5′‐extended transcript is the regulation by miRNA. Transcripts initiated at exon 1 have a distinct 5′‐UTRs region than those of previously reported HLA‐G transcripts. This may impact the regulation by miRNA and their cross‐talk with other miRNA acting on 3′‐UTRs. Disrupting this link may have substantial repercussion in the outcome of tumor cells (Lee *et al*., 2009; Manaster *et al*., 2012; Xie *et al*., 2005).

**Figure 9:**
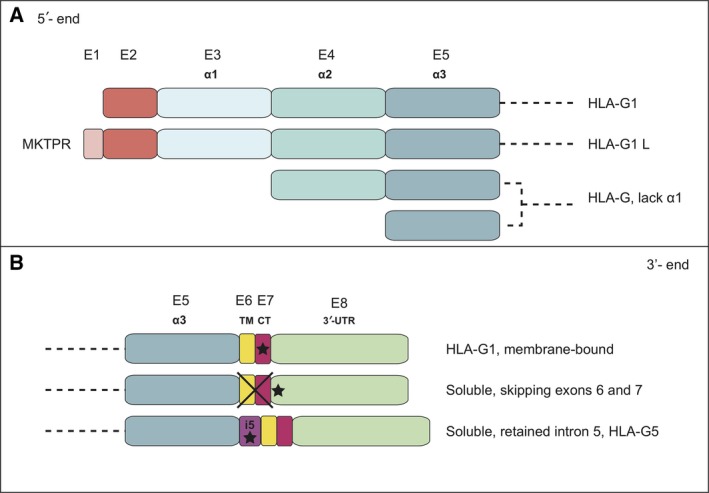
Diagrammatic representation of potentially expressed HLA‐G isoforms generated by alternative spliced transcripts. (A) N‐terminal ends including the additional five‐amino acid region of HLA‐G1L and the absence of the α1 domain that might yield isoforms containing the α2α3 domains or only the α3 domain. (B) Diagrammatic representation of isoforms with potentially different C‐terminal ends.

The results of our RNAseq approach revealed unreported HLA‐G transcripts that may be generated from the interplay of differential exon skipping events. Notably, the skipping of exons 6 and 7 may generate shorter transcripts that would encode proteins that lack the transmembrane domain and the cytoplasmic tail. Resulting from this, soluble proteins that differ from the known soluble protein HLA‐G5, ‐6, and 7 by the presence of distinct C‐terminal ends may be generated (diagrammatically represented in Fig. 9). These molecules might have diverse functions, as is the case for vascular endothelial growth factor (VEGF), for which the only presence of six amino acids at the C‐terminal end yields a protein that moves from a proapoptotic level to an antiapoptotic stage (Nowak *et al*., 2010).

Our molecular approach is consistent with the expression of HLA‐G transcripts that lack the signal peptide and exon 3 which encodes the alpha‐1 domain. The absence of signal peptide implies that no signaling to the membrane would occur. The resulting isoform may localize to different compartments inside the cell where it might interact with endocytosed receptors, as previously described for the HLA‐G receptor KIR2DL4 (Rajagopalan and Long, 2012) or with yet unknown receptors as previously suggested (LeMaoult *et al*., 2013; Menier *et al*., 2008). Alternatively, conserving the signal peptide in the absence of the α1 domain might result in a modified spatial configuration of the α2α3 molecule in which the Cys147 located in the α2 domain may interact together to form homodimers at the surface of the cell. It was previously shown that the Cys42 present in the alpha 1 domain of HLA‐G1 can form Cys42–Cys42 or Cys42–Cys147 disulfide bonds, but not Cys147–Cys147 bonds (Gonen‐Gross *et al*., 2003). The Cys147–Cys147 homodimers that might be formed in the α1‐deleted α2α3 molecules would possess two receptor binding sites which may affect the specificity or modulate the affinity of such HLA‐G isoforms for their receptors. Recently, the group of Maenaka reported that HLA‐G2, which lacks the α2 domain, naturally forms a β2‐microglobulin‐free and nondisulfide‐linked homodimer having an overall structure that resembles that of the HLA class II heterodimer (Kuroki *et al*., 2017). These homodimers were shown to bind the LILRB/ILT receptors with slow dissociation and a significant avidity effect. We can hypothesize that the structure of the α1‐deleted HLA‐G isoform (α2α3) would be similar to that of HLA‐G2 (α1α3) reported by Maenaka, as the alpha 1 and alpha 2 domains are significantly similar. Future studies are now required to gain insights into the function of the different α1‐deleted HLA‐G isoforms. Importantly, no detection of any of these proteins, possessing only the α2α3 domains or only the α3 domain, has been possible for now, either by immunochemistry or by cytometry as most of the antibodies currently available (87G, G233, MEMG, 4H84) recognize an epitope located in the alpha 1 domain.

A high prevalence of intron retention events was also demonstrated. We confirm that reads aligned on introns 3 and 5 (known before as intron 2 and intron 4 according to the IMGT/HLA nomenclature) are the most abundant. In addition, we demonstrate the retention of introns 4 and 7, and at lower levels, introns 1 and 6. Our results are also consistent with the presence of several introns retained simultaneously in the same transcript. The presence of intron 1 was never assessed before because according to IMGT/HLA nomenclature, it was considered the promoter region. However, although all HLA‐G isoforms are presumed to be generated by alternative splicing of a common pre‐mRNA, it remains possible that they could be derived from separate mRNA that are transcribed from distinct and internal promoters. These have been previously reported for several genes such as phosphodiesterase 5A (PDE5A) and TP53 (Campolo *et al*., 2017; Lin *et al*., 2002; Surget *et al*., 2013).

The retention of introns in expressed isoforms suggests functional consequences on cellular activity (Braunschweig *et al*., 2014) and has now opened a completely new vision on gene expression. Conventional splicing of introns from pre‐mRNA transcripts occurs in the nucleus of cells. However, recent articles have shown that transcripts with intron‐retained sequences are found in the cytoplasm. It was suggested that such sequences may be a component of particular RNA (named ‘sentinel’) that serves to generate transcript variants within the cytoplasm as a source for RNA‐based secondary messages. Work from a number of groups using a variety of cell types is steadily identifying a large number of cytoplasmic transcripts that retain introns (Buckley *et al*., 2014). Such RNA, although constituting only a small fraction of the mRNA population, might have significant implications for the function of proteins. However, a threshold for transcript abundance that is functionally relevant may be very difficult to establish. As cytoplasmic splicing continues to emerge, this ‘sentinel RNA hypothesis’ and its functional consequences are likely to become an area of keen interest.

Based on these findings, HLA‐G may be considered as part of the group of genes that were lately shown to act through many different isoforms, even though for most of them, their specific function still remains to be determined. For instance, the human TP53 gene encodes at least 12 isoforms, which are produced in normal tissues or stress conditions through alternative initiation of translation, usage of alternative promoters, and alternative splicing (Aoubala *et al*., 2011; Khoury and Bourdon, 2010, 2011). These have N‐ and C‐terminal truncated regions and different transcription/translational start sites. In recent years, it became evident that the only manner for TP53 to play so many distinct roles in regulating cell fate in response to different stresses is by the differential regulation of the various TP53 isoforms. The correlation between isoform and biological pathway, however, is a key question that still waits for an answer.

Another example is the VEGF gene (Woolard *et al*., 2009). Whereas hundreds of publications have described VEGF as a proangiogenic factor, an alternative splicing event generates specific antiangiogenic forms (Nowak *et al*., 2010). This latter only differs from the others by a modification of the last six amino acids at the C‐terminal end of the protein (Guyot and Pages, 2015). A similar situation is reported for the human PDE5A gene from which three isoforms are produced that differ only in the N‐terminal region. Interestingly, the translation initiation codon of PDE5A3 could be the second in‐frame ATG in PDE5A1 and PDE5A2; hence, the predicted PDE5A3 protein would be an N‐terminally truncated form of PDE5A1 and PDE5A2. Promoter activities were detected upstream from the PDE5A1‐specific exon and in the intron preceding the PDE5A2‐specific exon. The upstream PDE5A promoter is expected to direct the expression of all three PDE5 isoforms, while the intronic PDE5A2 promoter would only allow the expression of the PDE5A2 isoform independent of the other two isoforms (Lin *et al*., 2002). No biochemical or physiological function has been yet attributed.

Despite the enormous body of experimental knowledge accumulated over more than 30 years on different aspects of gene expression, there still remain many fundamental, unanswered questions regarding the biology and functional significance of the different isoforms. Among these, a critical issue is the complete clarification, and quantitative characterization, of the degree of functional specificity or overlapping exhibited by the different isoforms in the various cell lineages and tissues where they may be simultaneously expressed.

In the new era of developing clinical trials guided by molecular features of the tumors, our molecular survey has revealed a novel landscape of HLA‐G. More specifically, we have identified still unrecognized transcripts that might encode novel membrane‐bound and soluble proteins. It still remains to be defined whether the potentially different proteins that might be encoded play specific or overlapping functional roles in physiological and pathological conditions.

The unexpected level of complexity emerging with the discovery of these unannotated transcripts and the molecules that might encode, strengthens the idea that the use of current commercial antibodies may bias diagnostic, signifying that a large campaign to develop new antibodies is ineluctable. The complexity of HLA‐G expression in ccRCC together with the extent of inter‐ and intratumor heterogeneity raises key questions regarding how tumors can be optimally sampled and what are the best targets to improve the clinical outcome of patients with ccRCC. Ultimately, the data also suggest that extensive efforts must be made in the validation of predictive isoforms and reveal an unexpected need to reevaluate treatment strategies.

## Data accessibility

Research data pertaining to this article is located at figshare.com: https://doi.org/10.6084/m9.figshare.5319364

## Author contributions

DTLR and EDC involved in conception and design of the study. JR and JV performed experiments. DTLR, EDC, NRF, JLM, JFD, VR, ET, and JR analyzed and interpreted the data. DTLR, VR, and JV wrote the manuscript.
